# Longitudinal assessment of cyst-like lesions of the knee and their relation to radiographic osteoarthritis and MRI-detected effusion and synovitis in patients with knee pain

**DOI:** 10.1186/ar3132

**Published:** 2010-09-15

**Authors:** Daichi Hayashi, Frank W Roemer, Zineb Dhina, C Kent Kwoh, Michael J Hannon, Carolyn Moore, Ali Guermazi

**Affiliations:** 1 Quantitative Imaging Center, Department of Radiology, Boston University School of Medicine, FGH Building 3rd Floor, 820 Harrison Avenue, Boston, MA 02118, USA; 2 Division of Rheumatology and Clinical Immunology, University of Pittsburgh School of Medicine, 3500 Terrace Street, Pittsburgh, PA 15261, USA; 3 Pittsburgh VA Healthcare System, University Drive, Pittsburgh, PA 15240, USA; 4 Texas Woman's University, 6700 Fannin Street, Houston, TX 77030, USA; 5 Formally affiliated with: The Beverage Institute, One Coca-Cola Plaza, Atlanta, GA 30313, USA

## Abstract

**Introduction:**

The purpose of the present study was to determine the prevalence of cystic lesions and cyst-like bursitides in subjects with frequent knee pain and to assess their relation to radiographic osteoarthritis (OA) severity; to describe bilaterality and size fluctuation of the lesions over 6 months; and to assess relations between the prevalence of synovium-lined lesions communicating with the joint capsule and severity of magnetic resonance imaging (MRI)-detected effusion and synovitis.

**Methods:**

One hundred and sixty-three subjects (total 319 knees) aged 35 to 65 with chronic, frequent knee pain were included. Imaging with 3 Tesla MRI was performed at baseline and 6-month follow-up with the same protocols as those used in the Osteoarthritis Initiative. Severity of radiographic OA was assessed using the Kellgren-Lawrence grade (0 to 4). Severity of effusion and synovitis was graded 0 to 3 based on the Whole Organ Magnetic Resonance Imaging Score system. The associations of cysts and cyst-like bursitides and severity of radiographic OA, MRI-detected effusion and synovitis were analyzed using logistic regression controlling for clustering by person. The Wilcoxon signed-rank test was used to determine whether there was a significant change in the size of lesions between baseline and follow-up.

**Results:**

At least one lesion (any type) was present in 222 (70%) knees. The most prevalent lesions were popliteal cysts (40%, 128/319), followed by subgastrocnemius bursitis (15%, 49/319) and proximal tibiofibular joint cysts (8%, 26/319). Bilateral lesions were seen in 49% of the subjects. Only popliteal cysts and subgastrocnemius bursitis showed a significant change in size (*P *< 0.001). No trend was observed between prevalence of any of the cyst-like lesions analyzed and the increasing radiographic OA severity. Increasing prevalence of subgastrocnemius bursitis was associated with increasing severity of effusion (*P *= 0.0072) and synovitis (*P *= 0.0033).

**Conclusions:**

None of the cyst-like lesions analyzed seems to be a marker of radiographic OA severity in knees with chronic frequent pain. Subgastrocnemius bursitis may be used as a marker of effusion/synovitis severity. Bilateral cyst-like lesions are relatively commonly observed in people with chronic knee pain.

## Introduction

Fluid-equivalent lesions of the knee joint consist of a variety of pathologies ranging from benign intra-articular fluid collections to those associated with inflammatory or degenerative arthritis, infection and malignancy [1-4]. These pathologies have historically been detected by arthrography [5] and ultrasound [6], and are often seen on routine magnetic resonance imaging (MRI) scans. MRI has emerged as the technique of choice for characterizing the nature of these lesions, which show fluid-equivalent hypointensity on T1-weighted images and hyperintensity on T2-weighted images. MRI also enables determination of their anatomical relationship with the joint and other surrounding tissues [4,7].

**Figure 1:**
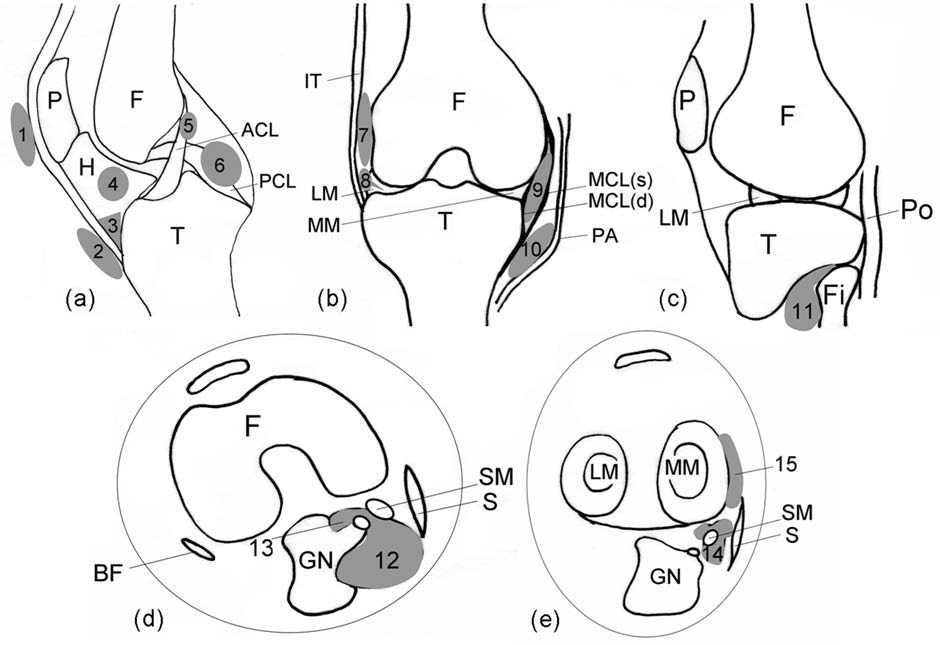
**Schematic diagram of the position of cysts and bursae assessed in the present study**. **(a) **Sagittal plane showing cruciate ligaments. **(b) **Coronal plane. **(c) **Sagittal plane showing tibiofibular joint. **(d) **and **(e) **Axial planes at the level of distal femur and menisci, respectively. These illustrations are intended to be a rough guide for readers to show approximate locations of these lesions and do not represent precise anatomical details. P, patella; H, Hoffa's fat pad; F, femur; T, tibia; ACL, anterior cruciate ligament; PCL, posterior cruciate ligament; IT, iliotibial band; LM, lateral meniscus; MM, medial meniscus; PA, pes anserinus; MCL(s), superficial layer of medial collateral ligament; MCL(d), deep layer of medial collateral ligament; Fi, fibula; Po, popliteus tendon; BF, biceps femoris tendon; SM, semi-membranosus tendon; GN, medial head of gastrocnemius muscle; S, sartorius muscle. Lesions: (1) prepatellar bursa; (2) superficial infrapatellar bursa; (3) deep infrapatellar bursa; (4) Hoffa's fat pad ganglion cyst; (5) ACL ganglion cyst; (6) PCL ganglion cyst; (7) iliotibial bursitis; (8) lateral meniscal cyst; (9) medial collateral ligament bursitis; (10) anserine bursa; (11) proximal tibiofibular joint cyst; (12) popliteal cyst; (13) subgastrocnemius bursa; (14) semi-membranous medial collateral ligament bursa; (15) medial meniscal cyst.

Detailed descriptions and illustrations of cyst-like lesions - that is, cysts and discrete fluid collections within bursae (bursitides) - have been published previously [4,7,8]. Bursae are synovium-lined structures usually not easily detected by any imaging method. Inflammation may result in a cyst-like appearance, however, due to accumulation of fluid within the bursa and thickening of the synovial membrane [8]. Locations of various bursae and cystic lesions of the knee are shown schematically in Figure 1.

To date, the prevalence of cysts and bursitides of the knee with or without symptoms has only been examined in a limited number of studies, which yielded discordant data [9,10]. Anserine, iliotibial, semi-membranosus medial collateral ligament (SM-MCL), and deep infrapatellar bursitides have each been shown to be more common in patients with knee osteoarthritis (OA) and pain in some studies [9,11]. In one study, however, deep infrapatellar bursitis showed an unusually high prevalence (41%) in asymptomatic knees [12]. Published information on the prevalence of bilateral cysts and bursitides of the knee in the setting of OA is limited [13].

The aim of our study was to assess the prevalence of cysts and cyst-like bursitides of the knee in a cohort of subjects with frequent knee pain and to examine their relation with radiographic OA severity. We also aimed to assess relations between synovium-lined cyst-like lesions communicating with the joint capsule and the severity of MRI-detected synovitis and effusion. Furthermore, we wished to evaluate whether the occurrence of these lesions was a bilateral phenomenon, and to describe their size changes over 6 months.

## Materials and methods

### Study sample

Subjects included in the present study were participants in the Joints On Glucosamine (JOG) cohort. The JOG study is a 6-month, double-blind, randomized controlled trial to examine the efficacy of oral glucosamine supplementation. Two hundred and one participants, aged 35 to 65 with mild to moderate chronic, frequent knee pain (Western Ontario and McMaster Universities score ≥25), were recruited at the University of Pittsburgh, PA, USA. Institutional Review Board approval and written informed consent from all participants were obtained for the present study.

### Types of fluid-filled lesions of the knee

**Figure 2:**
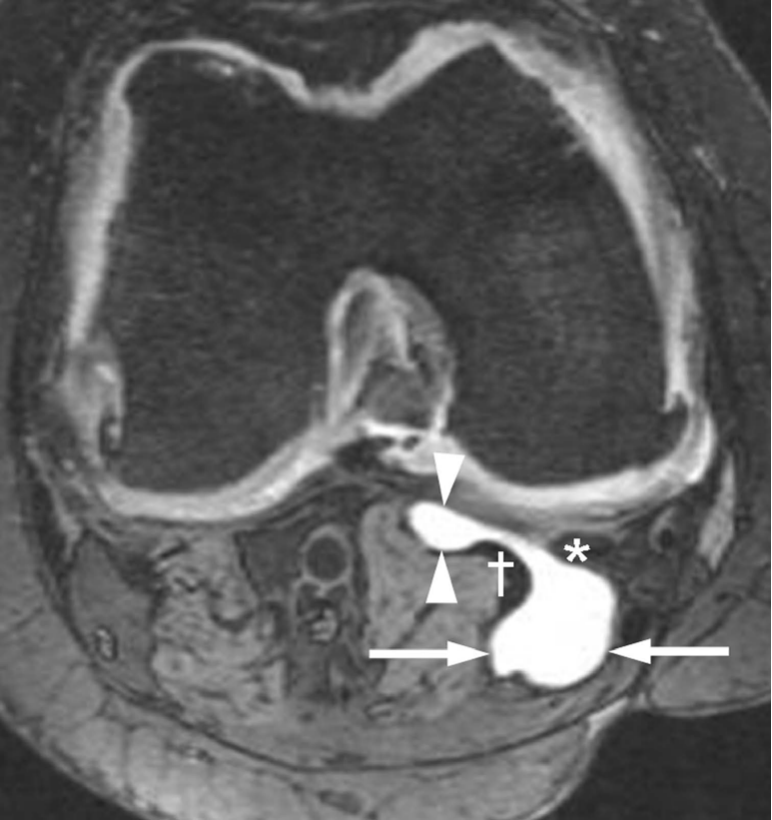
**Simultaneous presence of a popliteal cyst and subgastrocnemius bursitis in a right knee**. In this axial three-dimensional Dual Echo at Steady State image there is a communication through the gap between the medial head of gastrocnemius (†) and the semi-membranosus tendon (*). Arrows, popliteal cyst; arrowheads, subgastrocnemius bursitis.

Fifteen types of lesions were studied, including popliteal cysts (Figure 2), proximal tibiofibular joint (PTFJ) cysts, medial and lateral meniscal cysts, and anterior and posterior cruciate ligament (ACL and PCL) and Hoffa's fat pad ganglion cysts. In addition, the following bursitides with cyst-like appearances were included: anserine, prepatellar, superficial and deep infrapatellar, iliotibial, SM-MCL, medial collateral ligament, and subgastrocnemius bursitides [7] (Figures 1 and 2).

### Radiographic assessment of osteoarthritis

Radiographic images of the knee were obtained using a standard radiographic technique according to published guidelines [14]. Radiographs were read according to the Kellgren-Lawrence (K-L) grading scheme [15] by one expert musculoskeletal radiologist (AG) with 11 years of experience in K-L grading of knee radiographs. A random set of 30 radiographs (60 knees) were re-read to calculate the intraobserver reliability for the K-L grading of the knee, and the weighted kappa value was 0.89.

### Magnetic resonance imaging acquisition

Of 201 subjects in the JOG cohort, 38 were excluded because only a baseline MRI scan was available (24 subjects) or complete follow-up MRI assessment was not possible due to poor acquisition quality (14 subjects). Thus, 319 (161 left and 158 right) knees of 163 subjects were included in the present study.

MRI of each knee was performed using a 3 Tesla MRI (Siemens Trio, Erlangen, Germany) at baseline and at 6 months. The identical protocol used for the Osteoarthritis Initiative was applied in the JOG study, excluding the Fast Low Angle Shot sequence and the Multi-Echo Spin Echo T2 mapping sequence. Details of the full Osteoarthritis Initiative pulse sequence protocol and the sequence parameters have been published [16]. The protocol included the sagittal triplanar three-dimensional Dual Echo at Steady State sequence (slice thickness = 0.7 mm, interslice gap = 0 mm, repetition time = 16.3 ms, echo time = 4.7 ms, flip angle = 25°, field of view = 140 mm × 140 mm, matrix = 384 × 307 pixels, echo train length = 1, number of slices = 35, bandwidth = 185 Hz/pixel, number of excitations = 1, anterior/posterior phase encoding axis, acquisition time = 10 minutes 23 seconds) and the sagittal intermediate-weighted fat-suppressed sequence (slice thickness = 3 mm, interslice gap = 0 mm, repetition time = 30 ms, echo time = 3,200 ms, flip angle = 180°, field of view = 160 mm × 160 mm, matrix = 313 × 448 pixels, echo train length = 5, number of slices = 37, bandwidth = 248 Hz/pixel, number of excitations = 1, anterior/posterior phase encoding axis, acquisition time = 4 minutes 42 seconds). Axial and coronal images were reformatted from the sagittal three-dimensional Dual Echo at Steady State images.

### Magnetic resonance imaging assessment

One musculoskeletal radiologist (ZD) with 15 years of clinical experience, blinded to clinical data but not to the timepoint of the MRI examination, reviewed all images. The images were evaluated for the presence or absence of cystic lesions at baseline and at 6 months. Measurements of the largest diameter of each lesion were made to the nearest millimeter using a manual caliper on a standard DICOM viewer (eFilm Workstation, version 2.0.0; Merge Healthcare, Milwaukee, WI, USA). For the cystic lesions, unidimensional measurement was made based on the assumption that the size of a lesion increases or decreases in the same direction for all three axes - see Revised Evaluation Criteria in Solid Tumors (RECIST) (version 1.1) for size measurement of solid tumors [17] (in RECIST 1.1, cystic tumors are also considered a measurable lesion). The longest diameter of each lesion was measured on the sequence that showed the lesion in its largest dimension using the same plane (axial or sagittal) at baseline and at follow-up.

Of the lesions listed earlier, it was only possible to make meaningful size measurements on popliteal cysts, meniscal cysts, anterior cruciate ligament and PCL ganglion cysts, prepatellar bursitis, superficial infrapatellar bursitis and subgastrocnemius bursitis. These lesions had approximately circular or elliptical shape and their shape remained similar at two timepoints, enabling us to make meaningful size comparisons based on the assumption described earlier. However, it was not possible to measure the size of other lesions due to their completely irregular or undefinable shape. For these lesions, only the presence or the absence was noted. Development of a new lesion at follow-up was treated as an increase in size, and complete resolution of a pre-existing lesion at follow-up was treated as a decrease in size for the purpose of our analysis.

Severity of synovitis and effusion at baseline was semiquantitatively assessed according to the Whole Organ Magnetic Resonance Imaging Score (WORMS) system [18], taking into account all available sequences. Synovitis was graded 0 to 3 at infrapatellar and intercondylar sites (0, none; 1, mild; 2, moderate; 3, severe), and the maximum grade at either site was recorded. Effusion was also graded 0 to 3 (0, none; 1, ≤33% of maximum potential distention of the synovial cavity; 2, 33 to 66% of maximum potential distention; 3, ≥66% of maximum potential distention).

Intraobserver reliability for the detection of cystic lesions and their size measurement was assessed on a random sample of 20 knee MRI scans with all acquired sequences, and was excellent (κ = 0.88 for detection of cystic lesions; intraclass correlation coefficient for their size measurement = 0.94). Assessment of interobserver reliability was performed on a random sample of 50 knees by a second expert musculoskeletal radiologist (AG) with 11 years of experience in semiquantitative analysis of knee OA. Agreement for detection of cystic lesions was high (κ = 1.00), and the intraclass correlation coefficient for size measurements on the same sample was 0.99.

### Definition of significant change of size

During our intraobserver and interobserver reliability exercises, it was noted that the maximum difference between two readings for the measurement of the same lesion (either at baseline or at follow-up) was 4 mm for the popliteal cyst and 1 mm for other measurable lesions. We thus considered that any changes within these ranges might be attributable to measurement error. We defined the significant change of size to be ≥5 mm for popliteal cyst and ≥2 mm for other measurable lesions.

### Statistical analysis

The K-L grading was used to categorize knees according to the severity of radiographic OA. When there were eight or more of a particular type of lesion, the association between K-L grade and the prevalence of those lesions was tested using logistic regression controlling for clustering by person [19,20]. For cyst-like lesions that are lined by synovium and known to have a communication with the joint capsule [4] (that is, popliteal cysts, subgastrocnemius bursitis and PTFJ cysts), we also analyzed whether there was a linear trend between their prevalence and increasing severity of MRI-detected effusion and synovitis using logistic regression controlling for clustering by person.

Significance in associations between prevalence of cyst-like lesions and categorical/continuous variables was determined using Fisher's Exact Test for gender, and a *t *test for age and body mass index. These analyses were also performed only for those lesions observed in eight or more knees.

Finally, the Wilcoxon signed-rank test was used to determine whether there was a significant change in the size of lesions between baseline and follow-up.

All statistical calculations were performed using SAS^® ^software (version 9.1 for Windows; SAS Institute, Cary, NC, USA), except for the logistic regression that was performed using STATA (version 11.0; Statacorp LP, College Station, TX, USA).

## Results

### Subject characteristics

The present study included 163 participants with a mean age of 52 years (range 35 to 65 years, standard deviation ± 6) and a mean body mass index of 29 ± 4 kg/m^2^. Subjects were predominantly white (92%, 150/163) and approximately one-half were women (46%, 75/163). There were no statistically significant trends in the prevalence of any type of lesions with respect to age, gender or body mass index (data not shown), except for PCL ganglion cysts. Nine subjects (82%) who had PCL ganglion cysts were women, whereas 66 (43%) of those without the lesion were women (*P *= 0.024).

Characteristics of subjects who were excluded from the present study were similar to those included in terms of baseline presence of radiographic OA (that is, proportion of K-L grade ≥2 was 59% vs. 59%) and the presence of effusion (WORMS grade ≥1, 41% vs. 44%), but those excluded had lower prevalence of synovitis than those included (WORMS grade ≥1, 37% vs. 62%; *P *= 0.01, Fisher's exact test).

### Prevalence of cyst-like lesions and their relation to radiographic osteoarthritis

Table 1 summarizes the prevalence of all lesions. At least one fluid-filled lesion of any type was present in 208 knees (65%). The most prevalent type was popliteal cysts (Figure 2), found in 128 knees (40%). Subgastrocnemius bursitis, PTFJ cysts and Hoffa's fat pad ganglion cysts were observed in 49 knees (15%), 26 knees (8%) and 25 knees (8%), respectively. Nineteen (39%) subgastrocnemius bursitides co-existed with a communicating popliteal cyst (Figure 2). Other types such as SM-MCL bursitis, ACL and PCL ganglion cysts, and medial meniscal cysts were observed less frequently. Superficial and deep infrapatellar, prepatellar, anserine, and iliotibial bursitides were rarely observed but seemed to be more common in knees with OA than in those without OA. No lateral meniscal cysts were detected in any knee at baseline but one lesion had appeared at 6-month follow-up.

**Table 1:** Distribution of prevalent cysts and bursitides according to baseline Kellgren-Lawrence grades of radiographic osteoarthritis

**Type of lesion**^**a**^	Kellgren-Lawrence grade					
	
	All (*N *= 319)	0 (*N *= 103)	1 (*N *= 27)	2 (*N *= 37)	3 (*N *= 136)	4 (*N *= 16)
Any type*	208 (65)	56 (54)	19 (70)	29 (78)	90 (66)	14 (88)
Popliteal cyst	128 (40)	31 (30)	16 (59)	15 (41)	56 (41)	10 (63)
Subgastrocnemius bursitis	49 (15)	8 (8)	5 (19)	10 (27)	23 (17)	3 (19)
proximal tibiofemoral joint cyst	26 (8)	10 (10)	1 (4)	5 (14)	9 (7)	1 (6)
Hoffa's fat pad ganglion cyst	25 (8)	6 (6)	2 (7)	7 (19)	9 (7)	1 (6)
SM-MCL bursitis	13 (4)	1 (1)	0 (0)	1 (3)	7 (5)	4 (25)
PCL ganglion cyst	11 (3)	2 (2)	0 (0)	4 (11)	4 (3)	1 (6)
ACL ganglion cyst	9 (3)	3 (3)	0 (4)	2 (5)	4 (3)	0 (0)
Medial meniscal cyst	8 (3)	3 (3)	1 (4)	0 (0)	4 (3)	0 (0)
MCL bursitis	8 (3)	1 (1)	0 (0)	0 (0)	5 (4)	2 (13)
Prepatellar bursitis	6 (2)	0 (0)	0 (0)	0 (0)	5 (4)	1 (6)
Anserine bursitis	4 (1)	0 (0)	1 (4)	1 (3)	2 (2)	0 (0)
Iliotibial bursitis	3 (1)	0 (0)	1 (4)	0 (0)	0 (0)	2 (13)
Superficial infrapatellar bursitis	3 (1)	1 (1)	0 (0)	0 (0)	2 (2)	0 (0)
Deep infrapatellar bursitis	2 (1)	0 (0)	0 (0)	0 (0)	1 (1)	1 (6)
Lateral meniscal cyst	0 (0)	0 (0)	0 (0)	0 (0)	0 (0)	0 (0)

Data presented as number of lesions, either grand total or subtotal for each type of cystic lesions (percentage of knees that had a cystic lesion out of total 319 knees surveyed). *N*, number of knees that belong to each Kellgren-Lawrence grade; SM-MCL, semi-membranosus medial collateral ligament; PCL, posterior cruciate ligament; ACL, anterior cruciate ligament; MCL, medial collateral ligament. ^a^In order of descending frequency at baseline. *Statistically significant increasing prevalence with increasing Kellgren-Lawrence grade (*P *= 0.014, by logistic regression controlling for clustering by person).

Of the 319 knees, 103 (32%) were K-L grade 0, 27 (9%) were K-L grade 1, 37 (12%) were K-L grade 2, 136 (43%) were K-L grade 3, and 16 (5%) were K-L grade 4 (Table 1). A linear trend was found between increasing prevalence of fluid-filled lesions of any type and increasing K-L grade (K-L 0 = 54%, K-L 1 = 70%, K-L 2 = 78%, K-L 3 = 66% and K-L 4 = 88%; *P *= 0.014). This trend, however, did not extend to any individual type of lesions.

### Trend between prevalence of synovium-lined cyst-like lesions and severity of effusion and synovitis

Increasing prevalence of subgastrocnemius bursitis was associated with increasing severity of effusion (*P *= 0.0072) and synovitis (*P *= 0.0060) after controlling for clustering by person (Table 2). For popliteal cysts and PTFJ cysts, such a trend was not observed.

**Table 2:** Distribution of prevalent cysts according to baseline severity of effusion and synovitis

Type of lesion	WORMS grade			
	
	0	1	2	3
Effusion	*N *= 179	*N *= 80	*N *= 50	*N *= 10
Popliteal cyst	74 (41)	34 (43)	15 (30)	5 (50)
Subgastrocnemius bursitis*	20 (11)	16 (20)	8 (16)	5 (50)
Proximal tibiofemoral joint cyst	13 (7)	7 (9)	4 (8)	2 (20)
Synovitis	*N *= 121	*N *= 128	*N *= 61	*N *= 9
Popliteal cyst	41 (33)	59 (46)	25 (41)	3 (33)
Subgastrocnemius bursitis**	11 (9)	21 (16)	15 (25)	2 (22)
Proximal tibiofemoral joint cyst	8 (7)	12 (9)	4 (7)	2 (22)

Data presented as number of each type of lesion (percentage). WORMS, Whole Organ Magnetic Resonance Imaging Score; *N*, number of knees that belong to each WORMS grade. Significant increasing trend observed in subgastrocnemius bursitis: *P = 0.0072, **P = 0.0060.

### Size changes of cyst-like lesions over 6 months

Changes in the size of the lesion were significant only for popliteal cysts and subgastrocnemius bursitides (*P *< 0.001 for both) (Table 3). Of the 128 popliteal cysts identified at baseline, 95 (74%) exhibited a change in size over 6 months. Twenty-three increased in size (including the development of six new lesions) and 39 decreased in size. Of the 49 subgastrocnemius bursitides, 13 (27%) exhibited a significant change in size over 6 months (seven increased and six decreased). The remaining lesions did not show notable fluctuation in size.

**Table 3:** Comparison of the prevalence of cysts and bursitides at baseline and at 6-month follow-up

**Type of lesion**^**a**^	Prevalence (*N *= 319) (V1)	Incident lesion (V2)	**Increase in size (V2)**^**b**^	**Decrease in size (V2)**^**c**^
Popliteal cyst^d^*	128 (40)	6	23	39
Subgastrocnemius bursitis^d^*	49 (15)	0	7	6
PTFJ cyst	26 (8)	0	0	0
HFP ganglion cyst	25 (8)	0	0	1
SM-MCL bursitis	13 (4)	0	0	1
PCL ganglion cyst^d^	11 (4)	0	0	1
ACL ganglion cyst^d^	9 (3)	0	1	1
Medial meniscal cyst^d^	8 (3)	0	1	1
MCL bursitis	8 (3)	0	0	0
Prepatellar bursitis	6 (2)	0	2	0
Anserine bursitis	4 (1)	0	0	0
Iliotibial bursitis	3 (1)	1	1	1
Superficial infrapatellar bursitis^d^	3 (1)	0	0	0
Deep infrapatellar bursitis	2 (<1)	0	0	0
Lateral meniscal cyst^d^	0 (0)	1	1	0

Data presented as number of lesions, either grand total or subtotal for each type of cystic lesion (percentage of knees that had a cystic lesion out of total 319 knees surveyed). V1, visit at baseline; V2, visit at 6-month follow-up; *N *= total number of knees; PTFJ, proximal tibiofibular joint; HFP, Hoffa's fat pad; SM-MCL, semi-membranosus medial collateral ligament; PCL, posterior cruciate ligament; ACL, anterior cruciate ligament; MCL, medial collateral ligament. ^a^In order of descending frequency at baseline. ^b^The count for increase in size includes incident lesions seen at V2. ^c^The count for decrease in size includes lesions present at V1 but absent at V2. ^d^Meaningful size measurements were performed in these lesions only. A significant size change was defined to be ≥5 mm change for popliteal cysts and ≥2 mm for other lesions. For lesions without size measurements, increase in size means appearance of a new lesion at V2 and decrease in size means complete resolution of a pre-existing lesion at V2. *Significant rate of change in size between baseline and follow-up (*P *< 0.001, by Wilcoxon signed-rank test).

### Bilaterality

Seventy-six (49%) of the 156 subjects with readings for both knees exhibited at least one fluid-filled lesion (of any type) in both knees (Table 4). Bilateral popliteal cysts were observed in 34 subjects (22%) and bilateral subgastrocnemius bursitis in 11 subjects (7%). Bilaterality was observed to a lesser extent for PTFJ cysts, Hoffa's fat pad ganglion cysts, medial meniscal cysts and SM-MCL bursitis.

**Table 4:** Prevalence of bilateral cysts and bursitides at baseline

**Type of lesion**^**a**^	Bilateral (*N *= 156)
Any type	76 (49)
Popliteal cyst	34 (22)
Subgastrocnemius bursitis	11 (7)
proximal tibiofibular joint cyst	5 (3)
Hoffa's fat pad ganglion cyst	2 (1)
Medial meniscal cyst	1 (1)
Semi-membranosus medial collateral ligament bursitis	1 (1)

Data presented as number of subjects with bilateral cystic lesions, either grand total or subtotal for each type of cystic lesions (%). *N*, number of subjects. ^a^In order of descending frequency.

## Discussion

Our study showed that popliteal cysts, subgastrocnemius bursitis, PTFJ cysts and Hoffa's fat pad ganglion cysts were a common finding in painful knees. Other cyst-like lesions were infrequently observed (prevalence <5%). A linear trend can be shown between increasing prevalence of fluid-filled lesions of any type and increasing severity of radiographic OA, as assessed by K-L grade. No such trend, however, was demonstrated for individual type of lesions. An increasing prevalence of subgastrocnemius bursitis was associated with increasing severity of both effusion and synovitis, while that of popliteal and PTFJ cysts was not.

We observed that a large number of popliteal cysts changed in size at 6-month follow-up. These are the most frequently encountered cystic lesions of the knee, with a prevalence of 9% among older individuals with asymptomatic OA [10] and 33% for those with symptomatic OA [10]. Our finding most probably reflects the fact that the popliteal cyst is not a closed structure; that is, the cyst has a communication with the knee joint capsule [21,22], and the amount of fluid it contains may be affected by the degree of joint effusion and the intra-articular pressure. The increased pressure is thought to enlarge the popliteal cyst [23], and decreased pressure to reduce the size. The presence of popliteal cysts has been reported to be associated with joint effusion [21], and the presence of joint effusion is correlated with synovitis in knee OA [24]. We therefore expected to see a higher prevalence of popliteal cysts with higher effusion and synovitis grades on MRI scan. This trend was not observed, however, and the prevalence of poplitial cysts was high (30 to 50%) at all grades of effusion or synovitis (including grade 0) in our study. These findings suggest that the presence of popliteal cysts is equally common irrespective of effusion or synovitis status, and thus the presence of popliteal cysts on its own may not act as a marker of effusion/synovitis severity in subjects with chronic knee pain.

Another lesion that showed a significant change in size was subgastrocnemius bursitis. This bursa is located deep in the medial gastrocnemius muscle and commonly communicates with the semi-membranosus medial gastrocnemius bursa [25]. Subgastrocnemius bursa is therefore commonly seen to contain fluid on MRI scanning in patients with a popliteal cyst [21,26]. In contrast to popliteal cysts, there was a linear trend between the increasing prevalence of subgastrocnemius bursitis and the severity of effusion and synovitis. This discrepancy can be attributed to the fact that subgastrocnemius bursitis has a lower prevalence in knees with low grades of effusion or synovitis; that is, the prevalence of subgastrocnemius bursitis was similar to that of popliteal cysts in knees with grade 3 effusion (50% vs. 50%) or synovitis (22% vs. 33%), but was much lower in knees with grade 0 effusion (11% vs. 41%) or synovitis (9% vs. 33%). In contrast to popliteal cysts, therefore, subgastrocnemius bursitis may serve as an indirect marker for severity of synovitis and effusion.

Some authors treat subgastrocnemius bursitis as part of a popliteal cyst due to the presence of a communication between them, but our data imply that these two lesions should be treated as separate entities because of a discrepancy in their relation to increasing severity of effusion and synovitis. In the present study, although 19 knees had both of these lesions at the same time, simultaneous changes in size in the same direction rarely occurred (5%, 1/19). This may be due to the fact that the fluid can move between the two lesions depending on the patient's position or on the status of muscle contraction/relaxation.

There is conflicting evidence regarding the association of popliteal cysts with OA. Chatzopoulos and colleagues reported a higher prevalence of popliteal cysts (37%, 72/195) in knees with OA than in those without OA (2%, 1/54) [6]. In contrast, Tschirch and colleagues reported a 25% (26/102) prevalence of popliteal cysts in knees without OA, and suggested that the prevalence was similar in knees with or without OA [12]. Our results seem to be in general agreement with those of the latter study, but our reported prevalence is higher in knees without OA (30%, 31/103 knees with K-L grade 0). This variability might be attributable to differences in population size, imaging technique, and/or the detection limits employed by each investigator. Communications between the semi-membranosus medial gastrocnemius bursa and the knee joint are also known to be present in approximately one-half of knees without pain or OA [27]. This fact might explain the high prevalence of popliteal cysts in knees without radiographic OA in our study, and this speculation is supported by our data in which effusion was present in 30% (39/130) of knees without radiographic OA.

A popular theory for PTFJ cysts formation is that an increase in intra-articular pressure, possibly due to active synovitis or joint injury, causes an outpouching of the tibiofibular joint capsule herniating to form the synovial cyst [4,28]. We therefore expected to see increased prevalence in knees with higher effusion/synovitis grades. Although we could not demonstrate a statistically significant trend - most probably because only a small number of lesions were available for analysis - the prevalence of PTFJ cysts was higher in grade 3 effusion compared with grade ≤2 effusion (20% vs. 7 to 9%), and also higher in grade 3 synovitis compared with grade ≤2 synovitis (22% vs. 7 to 9%). A further study with a larger sample size may demonstrate a significant trend, but considering their lower prevalence compared with popliteal cysts and subgastrocnemius bursitis, PTFJ cysts are probably less useful as a marker of effusion/synovitis severity.

We have found that bilaterality of cysts or bursitides of the knee is a common occurrence, as more than one-half of the subjects exhibited bilaterality. The present study is the first to report such high prevalence of bilaterality but its clinical significance in management of knee OA remains uncertain.

There are several limitations in our study. First, studies have shown that not all fluid-containing bursae represent bursitis [12,29]. Because we did not have surgical or pathologic confirmation, some of the lesions we called bursitis might have been a simple collection of fluid without bursitis. Second, the shape of some of the fluid-filled lesions we studied could not be defined to make meaningful size measurements. Even the lesions for which size measurements were possible might not have strictly adhered to the assumption we made based on the revised RECIST 1.1. We attempted to minimize inaccuracies in our analysis by restricting size measurements to lesions that had well-defined circular or elliptical shapes without obvious distortive changes of the overall contour of the lesion between baseline and follow-up, and by incorporating a sufficiently large margin of error for the measurement. Third, the majority of cysts and bursitides occurred so infrequently that we could not perform statistical analysis for those lesions. A future study with a larger sample size will be required to confirm our findings and address these limitations. Last, our longitudinal analysis was limited to one follow-up assessment at 6 months. A longer follow-up period might have demonstrated size changes of lesions other than popliteal cysts and subgastrocnemius bursitis.

## Conclusions

In summary, various types of cysts and cyst-like bursitides of the knee joint are a common finding on MRI scans - with differing prevalence among cyst types, but with popliteal cysts being the most common lesion. None of the cyst-like lesions analyzed, however, seems to be a marker for radiographic OA severity in knees with chronic frequent pain. Popliteal cysts were commonly seen irrespective of effusion/synovitis grades. Subgastrocnemius bursitis may be used as a marker of effusion/synovitis severity, but popliteal or PTFJ cysts seem less useful for that purpose. Bilateral lesions were a relatively common occurrence and this phenomenon may warrant further studies, particularly to determine whether pre-emptive imaging of the contralateral knee has a place in clinical management of patients with knee pain.

## Abbreviations

JOG: Joints on Glucosamine; K-L: Kellgren-Lawrence; MRI: magnetic resonance imaging; OA: osteoarthritis; ACL: anterior cruciate ligament; PCL: posterior cruciate ligament; PTFJ: proximal tibiofibular joint; RECIST: Response Evaluation Criteria In Solid Tumors; SM-MCL: semi-membranosus medial collateral ligament; WORMS: Whole Organ Magnetic Resonance Imaging Score.

## Competing interests

AG received grants from General Electric Healthcare and National Institutes of Health. AG is the President of Boston Imaging Core Lab (BICL), LLC and is a consultant to MerckSerono, Facet Solutions, Genzyme and Stryker. FWR is a stockholder of BICL. CKK received funding from AstraZeneca and the Beverage Institute.

## Authors' contributions

DH, ZD, FWR, CKK, MJH, CM and AG contributed to the study concepts and design. DH, ZD, FWR, CKK, MJH and AG contributed to the literature search. DH, ZD, FWR, CKK, CM and AG contributed to the execution of this study, including participant recruitment, data acquisition and interpretation and analysis of images. CKK and MJH contributed to the statistical analysis. DH, ZD, FWR, CKK, MJH, CM and AG contributed to manuscript preparation and editing, and gave final approval for publication of this article. AG is the guarantor of the integrity of the entire study.
